# Inhibition of α-Glucosidase Activity and Islet Amyloid PolyPeptide Fibril Formation by *Rubus* *ulmifolius* Fruit Extract: A Potential Therapeutic Approach for Type 2 Diabetes Mellitus

**DOI:** 10.3390/plants14213247

**Published:** 2025-10-23

**Authors:** Sonia Floris, Barbara Noli, Cristina Cocco, Antonella Fais, Benedetta Era, Carlo Ignazio Giovanni Tuberoso, Valentina Masala, Franca Piras, Valeria Sogos, Amalia Di Petrillo, Antonio De Agostini, Francesca Pintus, Cinzia Sanna

**Affiliations:** 1 Department of Life and Environmental Sciences, Biomedical Section, University of Cagliari, 09042 Cagliari, Italy; s.floris@unica.it (S.F.); fais@unica.it (A.F.); era@unica.it (B.E.); tuberoso@unica.it (C.I.G.T.); valentina.masala2@unica.it (V.M.); 2 Department of Biomedical Sciences, University of Cagliari, 09042 Cagliari, Italy; barbara.noli@unica.it (B.N.); cristina.cocco@unica.it (C.C.); fpiras@unica.it (F.P.); sogos@unica.it (V.S.); 3 Department of Medical Science and Public Health, University of Cagliari, 09042 Cagliari, Italy; amalia.dip@unica.it; 4 Department of Life and Environmental Sciences, Botany Section, University of Cagliari, Via Sant’Ignazio da Laconi 13, 09123 Cagliari, Italy; antonio.dea@unica.it (A.D.A.); cinziasanna@unica.it (C.S.)

**Keywords:** wild blackberries, IAPP, type-2 diabetes, α-glucosidase, caspase 1, functional food

## Abstract

This study examines the antidiabetic potential of *Rubus ulmifolius*, a shrub traditionally used for medicinal and nutritional purposes. The aim was to assess the fruit extract’s inhibitory effect on α-amylase and α-glucosidase, therapeutic targets in type 2 diabetes (T2D), given their role in carbohydrate digestion. Considering the role of islet amyloid polypeptide (IAPP) aggregation in pancreatic β-cell dysfunction in T2D, the extract’s effect on inhibiting this process was also evaluated. Cytocompatibility and antioxidant effects were tested in Caco-2 cells, while caspase-1 activity was measured to evaluate anti-inflammatory potential. Phytochemical analysis of *R. ulmifolius* fruits revealed various phenolic compounds, with anthocyanin as the most abundant one. The cyanidin-3-*O*-glucoside accounted for 86% of all anthocyanins. Among flavonoids, the most represented ones were quercetin-HMG-glucoside and a kaempferol derivative, while ellagic acid glucuronide was the predominant ellagitannin. The extract showed significantly higher α-glucosidase inhibition with an IC_50_ value of 2.8 µg/mL, 32 times more effective than acarbose, and it markedly inhibited IAPP aggregation in a dose-dependent manner. It demonstrated antioxidant activity in cellular and acellular systems, without cytotoxicity. Caspase-1 activity was also reduced in intestinal cells. These findings support *R. ulmifolius* fruits as a promising functional food for managing T2D and preserving pancreatic function.

## 1. Introduction

Type 2 diabetes mellitus (T2D) is a chronic metabolic condition involving reduced insulin sensitivity and disrupted blood glucose regulation. As of 2022, approximately 828 million adults worldwide are living with diabetes, and the global mortality rate due to diabetes remains high [1]. Hyperglycemia is associated with long-term complications such as cardiovascular disease, retinopathy, neuropathy, and kidney damage, as well as an increased risk of infections. Persistent hyperglycemia promotes oxidative stress and inflammatory pathways, which are key contributors to the development of diabetic complications. Controlling postprandial glucose levels in T2D often involves targeting digestive enzymes such as α-amylase and α-glucosidase to slow carbohydrate breakdown. α-Amylase (EC 3.2.1.1) breaks starch down into oligosaccharides, which are further hydrolyzed by α-glucosidases. α-Glucosidase (EC 3.2.1.3) is a hydrolytic enzyme which cleaves the 1,4-α glycosidic bonds, producing free α-d-glucose which is absorbed into the bloodstream by intestinal cells, resulting in postprandial hyperglycemia. Inhibiting these enzymes slows glucose release into the bloodstream, promoting better glycemic control and helping prevent complications linked to T2D.

Another critical pathological feature of T2D is the formation of amyloid plaques composed of islet amyloid polypeptide (IAPP). IAPP or amylin is a hormone released alongside insulin by pancreatic β-cells in response to increased blood glucose. Physiologically, IAPP plays a regulatory role in glucose metabolism by delaying gastric emptying and inducing satiety signals. However, in pathological conditions, IAPP can undergo misfolding and aggregation, forming toxic amyloid plaques leading to β-cell death. Preventing IAPP fibril formation has emerged as a potential strategy to preserve β-cell function in diabetic patients [2]. Furthermore, since T2D is characterized by oxidative stress, which is exacerbated by both hyperglycemia and amyloid-induced toxicity [3], antioxidant agents may complement strategies aimed at inhibiting α-amylase and α-glucosidase or preventing IAPP aggregation. These combined approaches could potentially provide a multi-target strategy in T2D treatment.

Chronic low-grade inflammation plays a key role in the pathophysiology of T2D, contributing to impaired insulin signaling and β-cell dysfunction through the persistent release of pro-inflammatory cytokines. Notably, studies in animal models have shown that inhibiting or knocking out Caspase-1 can improve insulin sensitivity and glucose tolerance, emphasizing its role as a possible therapeutic target in T2D [4].

Given the critical role of combined unhealthy lifestyle factors in T2D development, strategies emphasizing physical activity and a healthy diet are crucial for prevention.

Evidence suggests that certain foods, particularly those rich in bioactive compounds, such as quercetin, catechins, kaempferol, gallic acid, may assist in improving glycemic response and modulating oxidative pathways, thereby contributing to the prevention or delay of T2D and its related complications in high-risk individuals [5,6,7]. Fruit species with high levels of phytochemicals are being explored for their potential to contribute to chronic disease prevention, including metabolic disorders [8,9].

*Rubus ulmifolius* Schott (Rosaceae family) is a perennial spiny shrub indigenous to Europe and North America and widely distributed in Asia and North Africa [10]. *R. ulmifolius* is widely used in different Mediterranean traditional medicines: in Italy, its leaves are used for treating wounds, pimples, and skin diseases, as well as for managing gastric and urogenital inflammation, sore throat, diarrhea, menstrual pain, liver ailments, and hypertension [11,12]. Additionally, the leaves of *R. ulmifolius* are traditionally recognized for their hypoglycemic and antidiabetic properties [11,13]. They contain flavonoids such as kaempferol 3-*O*-rutinoside and naringenin, along with tannins, alkaloids, and steroids [14,15]. *R. ulmifolius* produces edible fruits (wild blackberries), commonly consumed fresh or made into juices, jams, syrups, and desserts in Mediterranean regions, and extensively used as ingredients in food and dietary supplements.

*R. ulmifolius* fruits are rich in beneficial primary and secondary metabolites, including carbohydrates, vitamins, phenolic compounds (mainly ellagic acid and cyaniding derivatives, gallic acid, quercetin and isoquercitrin), fibers, and minerals [10,16,17]. Research has highlighted their noteworthy biological activities, such as antioxidant [18], antimicrobial [16], anticancer [17,19] and neuroprotective effects [17]. However, little is known about their antidiabetic properties [20] and, to the best of our knowledge, no information on their capability to inhibit the aggregation of IAPP is available.

In this context, this study investigates, through a multitarget approach, the potential antidiabetic properties of *R. ulmifolius* fruits collected in Sardinia, Italy, by evaluating its inhibitory effects on α-amylase and α-glucosidase activities, its capacity to prevent islet amyloid polypeptide (IAPP) aggregation, and its antioxidant and anti-inflammatory properties in relevant in vitro models.

## 2. Results

### 2.1. α-Amylase and α-Glucosidase Inhibition

The inhibitory effects of *R. ulmifolius* fruit extract on α-glucosidase and α-amylase activities were investigated, and the results are summarized in Table 1. The extract demonstrated a remarkable α-glucosidase inhibition with an IC_50_ value of 2.80 ± 0.56 µg/mL, 32 times more effective than the standard drug acarbose. On the contrary, the *R. ulmifolius* fruit extract showed a lower affinity for the α-amylase enzyme, exhibiting a modest inhibition (IC_50_ value exceeding 100 µg/mL).

These findings underscore the selectivity of *R. ulmifolius* fruit extract for α-glucosidase inhibition, highlighting its role as a possible therapeutic agent for managing postprandial hyperglycemia.

**Figure 1:**
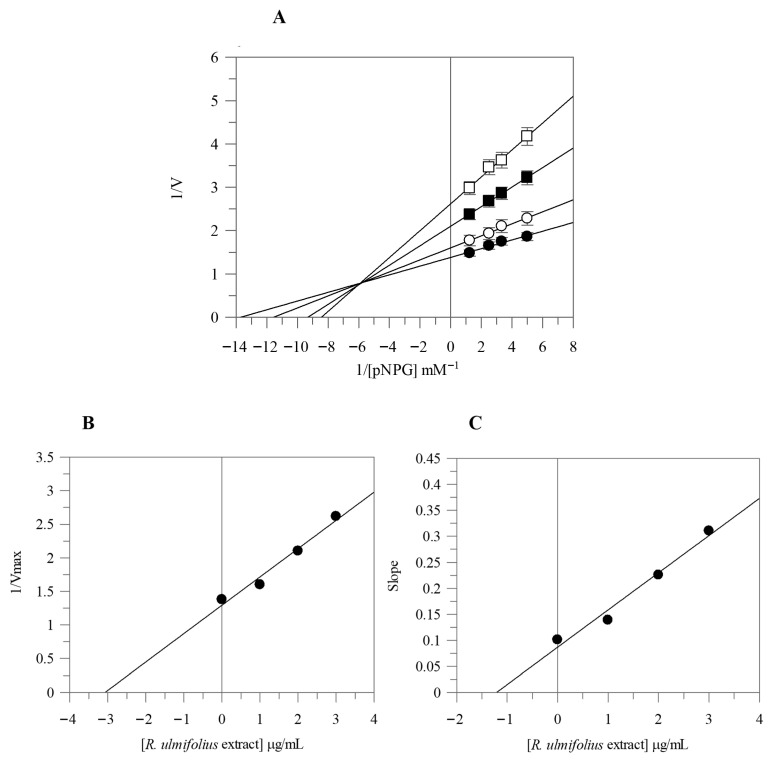
Kinetic analysis of the α-glucosidase inhibition by *R. ulmifolius* fruit extract. Lineweaver–Burk analysis (**A**) using extract concentrations of 0 (●), 1 (○), 2 (■), and 3 (□) µg/mL, and secondary plots (**B**,**C**). Data represents the mean ± standard deviation for three independent assays.

Due to the strong α-glucosidase inhibitory activity of the extract, we proceeded with its kinetic analysis. The Lineweaver-Burk plots of *R. ulmifolius* fruit extract exhibited characteristics of mixed type inhibition. In fact, as the extract concentration increased, the plots resulted in a series of straight lines with different slopes and y-intercepts which intersected in the second quadrant, as illustrated in Figure 1A. This kinetic behavior suggests that the extract can bind both to the free enzyme and to the enzyme-substrate complex. The equilibrium constants for inhibitor binding to the free enzyme (KI) and to the enzyme-substrate complex (KIS) were calculated from the slope (Km/Vmax) or from the 1/Vmax values (*y*-intercepts) plotted against inhibitor concentration, resulting as 1.19 µg/mL and 3.07 µg/mL, respectively (Figure 1B,C).

### 2.2. ABTS Radical Scavenging Activity and Determination of the Total Phenolics and Flavonoids

The antioxidant capacity of *R. ulmifolius* fruit extract was evaluated through the ABTS assay, with an EC_50_ value of 72.42 ± 0.62 µg/mL (Table 2).

The total phenolic and flavonoid contents suggest that these compounds may contribute to the observed antioxidant activity, since their content in the *R. ulmifolius* fruit extract was 28.69 ± 0.92 mg GAE/g dried weight (dw), and 5.12 ± 0.68 mg QE/g dw, respectively.

Collectively, these results highlight the antioxidant potential of *R. ulmifolius* fruit extract, although it is notably less potent than the standard Trolox, as evidenced by the higher EC_50_ value.

### 2.3. Cell Viability and Intracellular ROS Levels

**Figure 2:**
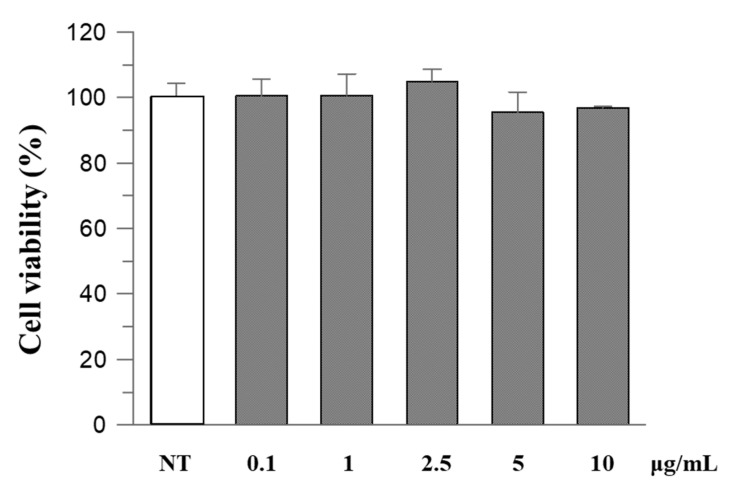
The effect of *R. ulmifolius* fruit extract on Caco-2 cell viability. Cells were treated with different concentrations of the extract and studied using the MTT assay. Data are expressed as a percentage of control. Data represent the mean (±standard deviation) of six independent experiments. Statistical analysis was performed and no significant differences were observed (ANOVA, *p* = 0.2913).

To evaluate the safety of *R. ulmifolius* fruit extract, Caco-2 cells were exposed to different concentrations of the extract for 24 h and then examined using the MTT test. The results indicated that the extract did not exhibit cytotoxic effects, and cell viability remained above 90% at the highest concentration (Figure 2).

**Figure 3:**
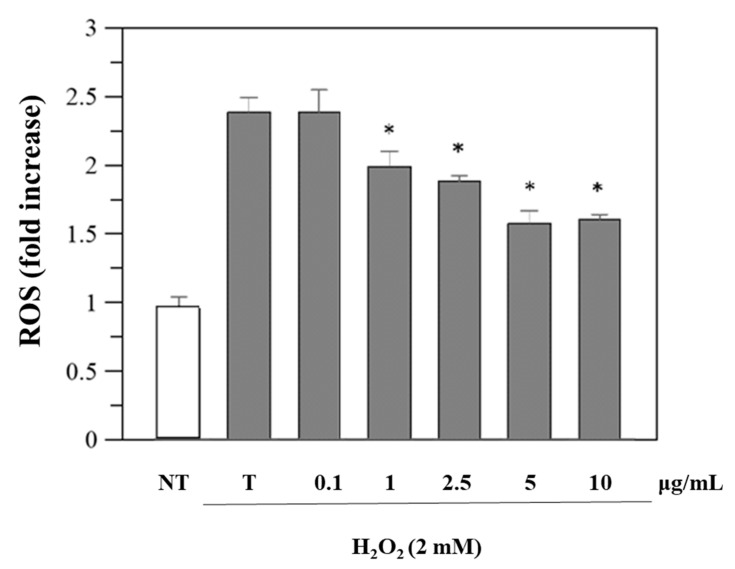
Inhibition of H_2_O_2_-induced ROS generation (1 h-incubation with 2 mM H_2_O_2_) by *R. ulmifolius* fruit extract on Caco-2 cells. NT, non-treated cells; T, cells treated with H_2_O_2_ only. Data represent the mean (±standard deviation) of six independent experiments. Asterisks indicate values statistically different from cells treated with H_2_O_2_ only (T), based on ANOVA (*p* < 0.0001) followed by Tukey’s post hoc test. The following *p*-values were obtained for the extract concentrations: 1 µg/mL (*p* = 0.0036), 2.5 µg/mL (*p* = 0.0004), 5 and 10 µg/mL (*p* < 0.0001).

Since viability was unaffected, we performed further cellular experiments using the same concentrations. Reactive oxygen species (ROS) levels in cells before and after exposure to oxidative stress and after treatment with *R. ulmifolius* fruit extract were evaluated. The study was conducted using DCFHDA, a compound which easily penetrates the cell membrane and is subsequently hydrolyzed by the intracellular esterases into DCFH. An increase in DCF fluorescence indicates the oxidation of DCFH by intracellular ROS including H_2_O_2_. As illustrated in Figure 3, the extract reduces H_2_O_2_-induced ROS generation in a dose–response manner. These findings indicate that *R. ulmifolius* fruit extract may reduce ROS formation within cells, pointing to its potential as a protective agent against oxidative damage, a common concern in diabetic patients due to the prolonged high blood glucose levels and the associated free radical production. Moreover, as shown in Figure S1, pre-treatment with different concentrations of the extract also significantly preserves cell viability against H_2_O_2_-induced cytotoxicity.

Thus, this antioxidant property of the extract is particularly important, given that oxidative stress is a key factor in the development of diabetes and its complications.

### 2.4. Modulation of Caspase-1 Activity by R. ulmifolius in Caco-2 Cells

**Figure 4:**
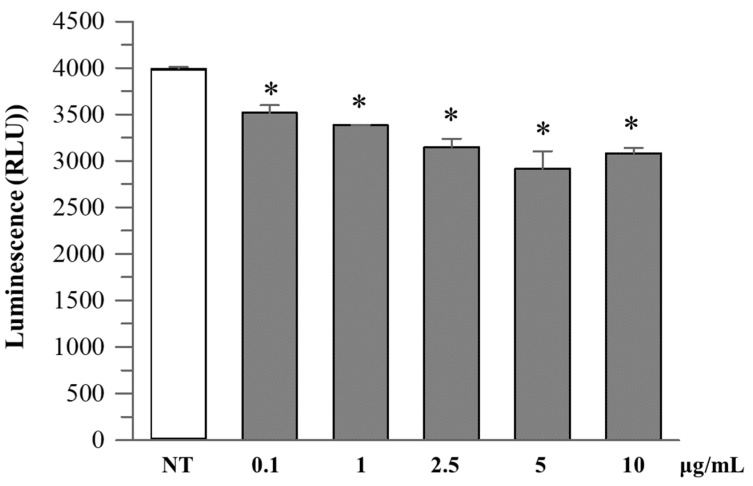
Caspase-1 activity in Caco-2 cultured cells. Caco-2 cells were treated with increasing concentrations (0.1–10 µg/mL) of *R. ulmifolius* extract for 24 h. Caspase-1 activity in cell lysates was measured using a luminescence-based assay. Data are presented as mean ± SD of two independent experiments. Asterisks indicate values statistically different from non-treated cells (NT) based on ANOVA (*p* = 0.0003) followed by Tukey’s post hoc test. The following *p*-values were obtained for the extract concentrations: 0.1 µg/mL (*p* = 0.0180), 1 µg/mL (*p* = 0.0051), 2.5 µg/mL (*p* = 0.0009), 5 µg/mL (*p* = 0.0002) and 10 µg/mL (*p* = 0.0006).

**Figure 5:**
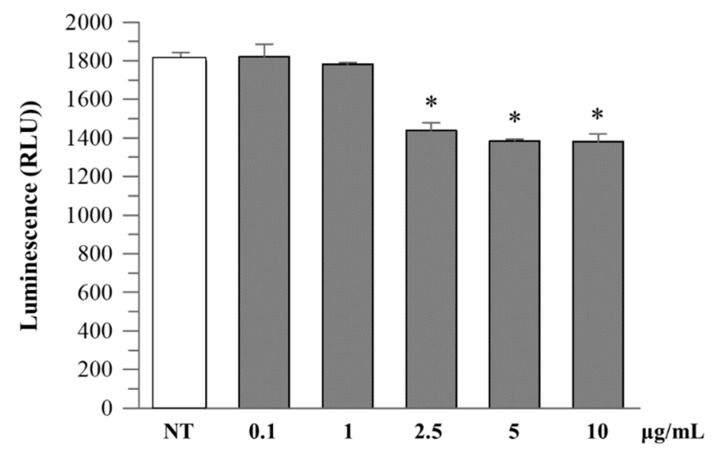
Caspase-1 activity in cell culture medium. Caco-2 cells were treated as described above, and extracellular caspase-1 activity was measured in the culture supernatant. A dose-dependent reduction in luminescence signal was observed at higher extract concentrations. Data are expressed as mean ± SD of two independent experiments. Asterisks indicate values statistically different from non-treated cells (NT) based on ANOVA (*p* < 0.0001) followed by Tukey’s post hoc test. The following *p*-values were obtained for the extract concentrations: 2.5 µg/mL (*p* = 0.0007), 5 and 10 µg/mL (*p* = 0.0003).

*R. ulmifolius* fruit extract treatment reduced caspase-1 activity in Caco-2 cells in a dose-dependent manner up to 10 µg/mL. At concentrations of 2.5–10 µg/mL, a significant decrease in luminescence signal was observed compared to the untreated control, suggesting suppression of inflammasome activation (Figure 4). Analysis of cell culture supernatants revealed that extracellular caspase-1 activity was also decreased with low-dose extract treatment (Figure 5), indicating a reduction in inflammatory caspase-1 release, potentially reflecting lower levels of pyroptosis or IL-1β maturation. These findings suggest that *R. ulmifolius* fruit extract may possess anti-inflammatory properties by targeting the inflammasome pathway.

### 2.5. Inhibition of IAPP Aggregate Formation

**Figure 6:**
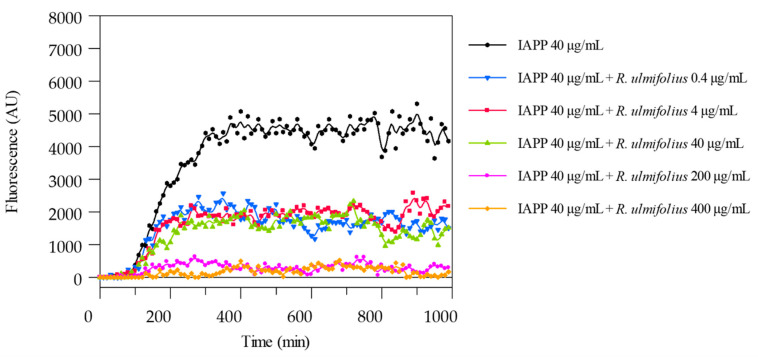
ThT fluorescence emission plots corresponding to β-sheet formation of IAPP in the presence of *R. ulmifolius* fruits extract.

To detect and measure the formation of IAPP amyloid fibrils, the ThT binding assay was used. The potential of *R. ulmifolius* fruit extract to inhibit fibril formation was investigated. The extract was added at different ratios to IAPP solutions and the kinetics of amyloid fibril formation were monitored by measuring ThT fluorescence emission. The extract prevented fibril formation at both the 1:5 (IAPP 40 μg/mL:extract 200 μg/mL) and 1:10 (IAPP 40 μg/mL:extract 400 μg/mL) ratio, as shown in Figure 6. In both cases, the corresponding curves are flat and superimposed, indicating the absence of fibril aggregation. By reducing the extract concentration, that is at 1:1 (IAPP 40 μg/mL:extract 40 μg/mL), 1:0.1 (IAPP 40 μg/mL:extract 4 μg/mL) and 1:0.01 (IAPP 40 μg/mL:extract 0.4 μg/mL) ratio, a quantitative reduction in IAPP formation is detected, indicating that lower concentrations of the extract may lead to a decrease in the amyloid fibrils formation and highlighting that the inhibition occurs in a dose-dependent manner. The ThT assay was performed in triplicate and monitored for up to 150 h.

In patients with T2D, circulating plasma concentrations of IAPP typically range from approximately 5 to 10 pmol/L under fasting or mildly stimulated conditions [21]. These physiological levels are several orders of magnitude lower than the 40 µg/mL (10 µM) IAPP concentration employed in our in vitro assays. Consequently, the extract:IAPP ratios tested (from 1:0.01 to 1:10) indicate that relatively low concentrations of the extract might achieve inhibitory efficacy in vivo, especially considering potential local accumulation of bioactive metabolites or enhanced extract bioavailability through optimized formulations. Thus, our observation of dose-dependent inhibition at extract:IAPP ratios as low as 1:0.01 supports the translational potential of the extract as an amyloidogenesis inhibitor under physiologically relevant conditions.

### 2.6. Quali-Quantitative Determination of Phenolic Compounds in R. ulmifolius Fruit Extract

The extract from *R. ulmifolius* fruits was qualitatively analyzed using (HR) LC-ESI-QToF MS/MS in both positive and negative ion mode, and quantification of targeted phenolic compounds was performed through HPLC-PDA analysis.

**Figure 7:**
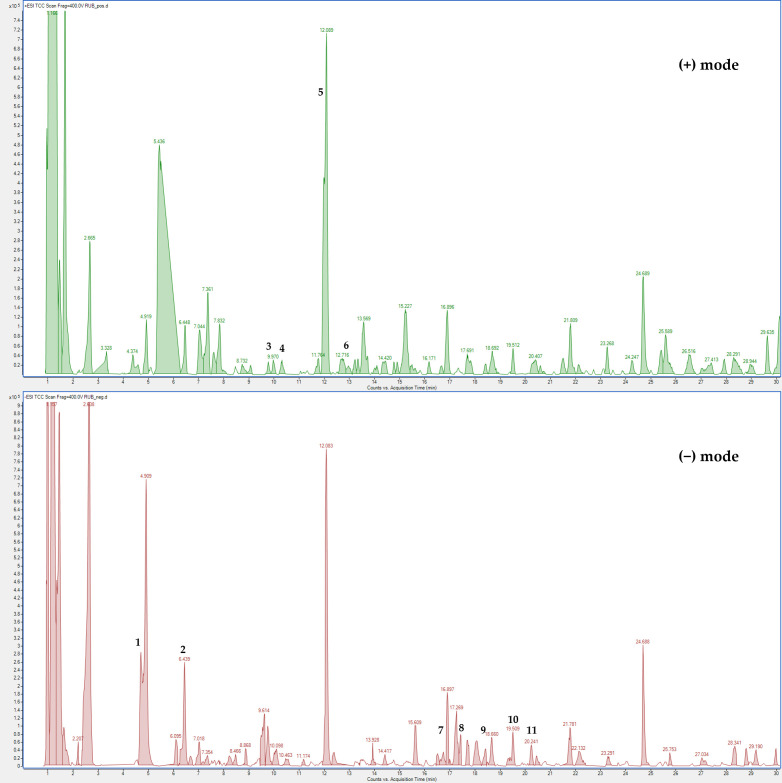
(HR) LC-ESI-QTOF MS Total Compound Chromatogram of *R. ulmifolius*  fruit extract acquired in positive and negative ion mode. Chromatographic conditions are described in the text. Peak identification (number) is given in Table S1.

The LC-MS profile in both ionization modes revealed numerous signals corresponding to molecular ions of phenolic compounds, with flavonoid derivatives representing the major constituents (Figure 7).

The positive LC-MS profile showed the presence of anthocyanins, which are not visible in negative ion mode. Individual components were identified by comparison of their *m*/*z* values in the total compound chromatogram (TCC) profile with those previously reported in the literature (Table S1). In addition, 12 compounds were identified through comparison of their experimental MS/MS spectra with known fragmentation patterns reported in the literature or with the fragmentation patterns and spectra available in a public mass spectral database [22,23,24]. Table S1 reports the identified compounds, organized by retention times, the chemical formula determined from accurate mass measurement, MS/MS data, the references used for identification, and the identification confidence levels [25].

Compound 1 was identified as protocatechuic acid based on the [M − H]^−^ at *m*/*z* 153.0443 and comparison with a pure standard; it was previously detected in other plants belonging to the Rosaceae family [26,27]. Peak 2 was attributed to chlorogenic acid due to the [M − H]^−^ at *m*/*z* 353.110 and a fragment at *m*/*z* 191.0777 (loss of a quinic acid unit) and the comparison with literature data [20,28]. Compound 3 was identified as cyanidin-3-*O*-glucoside with a molecular formula due to the [M + H]^+^ at *m*/*z* 449.1082 and a fragment at 287.0548 (loss of a cyanidin unit) and the comparison with pure standard and literature data [16,20,28]. Peak 4 was attributed to pelargonidin-3-*O*-glucoside with molecular formula C_21_H_21_O_10_+ due to the [M + H]^+^ at *m*/*z* 433.1114 and a fragment at 271.5544 and the comparison with literature data [16,20,28] and the comparison with pure standard. Peak 5 was attributed to cyanidin-3-*O*-xyloside with molecular formula C_20_H_19_O_10_+ due to the [M + H]^+^ at *m*/*z* 419.0972 and a fragment at 287.0546 (loss of a cyanidin unit) and the comparison with literature data [16,20,28]. Compound 6 was identified as cyanidin-3-*O*-dioxalyl-glucoside with molecular formula C_21_H_28_O_15_+ due to the [M + H]^+^ at *m*/*z* 593.2197 and a fragment at 287.0547 (loss of a cyanidin unit) and the comparison with literature data [16,20,28]. Peaks 7 and 9 were attributed to ellagic acid derivatives. Peak 7 was attributed to ellagic acid pentoside with molecular formula C_19_H_14_O_12_ due to the [M − H]^−^ at *m*/*z* 433.0634 and fragments at 301.0186 (loss of an ellagic acid unit) and 300.0116 and the comparison with previous studies [16]. Peak 9 was attributed to ellagic acid glucuronide with molecular formula C_20_H_14_O_14_ due to the [M − H]^−^ at *m*/*z* 477.0849 and fragments at 301.0560 (loss of an ellagic acid unit) and the comparison with literature data [16]. Compound 8 was identified as quercetin-3-*O*-rhamnoside due to the [M − H]^−^ at *m*/*z* 447.0774 and fragments at 300.0128 and 301.0196 (loss of quercetin unit) and the comparison with pure standard and literature data [20,28]. Compound 10 was identified as quercetin-hydroxymethylglutaryl (HMG)-glucoside with molecular formula C_27_H_18_O_16_ due to the [M − H]^−^ at *m*/*z* 607.1422 and fragments at 300.0404 and 301.0549 (loss of a quercetin unit) and the comparison with previous studies [16,20,29]. Compound 11 was tentatively identified as a kaempferol derivative with molecular formula C_21_H_20_O_11_ due to the [M − H]^−^ at *m*/*z* 447.1114 and fragments at 255.0539, 284.0536 and 285.05978 (loss of a kaempferol unit) and the comparison with literature data related to other species of the Rosaceae family [29,30,31]. Peak 12 was attributed to kaempferol-3-*O*-rutinoside due to the [M − H]^−^ at *m*/*z* 593.1423 and fragments at 284.0541 and 285.0603 (loss of a kaempferol unit) and the comparison with pure standard and literature data [20,28].

HPLC-PDA analysis confirmed the presence of anthocyanins detected by (HR) LC-ESI-QToF MS/MS in positive ion mode. The most abundant anthocyanin was cyanidin-3-*O*-glucoside (25.18 ± 1.76 mg/g dw) which accounted for 86% of all anthocyanins. Among flavonoids, the most represented were quercetin-HMG-glucoside and a kaempferol derivative, while ellagic acid glucuronide was the most abundant among the ellagitannins (1.35 ± 0.07 mg/g dw) (Table 3).

## 3. Discussion

The term “functional foods” refers to foods which have been scientifically demonstrated to provide potential health benefits. Any food containing biologically active components is considered functional due to its association with physiological advantages, particularly in preventing chronic diseases such as T2D. Regular consumption of functional foods has been shown to aid in glycemic control, regulation of blood pressure, and activation of antioxidant enzymes, thereby contributing to the prevention or delay of T2D and its related complications in high-risk individuals [5,6]. Among plant-based functional foods, fruit species are especially relevant due to their rich phytochemical profiles and associated health effects [8]. Among them, strawberries, blueberries, and blackberries are highly appreciated for their appealing color, desirable flavor, and taste. These fruits are rich in various classes of phytoconstituents, with phenolic compounds being primarily responsible for their beneficial health effects [32,33].

This study investigates the potential antidiabetic properties of a hydroalcoholic extract from wild blackberries (*Rubus ulmifolius* fruits) collected in Sardinia, Italy. Analytical profiling confirmed that *R. ulmifolius* fruit extract is rich in phenolic constituents, with anthocyanins being the predominant class, contributing to both color and biological activity. These findings align with previous studies [16,20]; however, quantitative differences in the identified compounds were observed by comparing them to literature reports. In particular, cyanidin 3-*O*-glucoside and pelargonidin-3-*O*-glucoside were the major anthocyanins in our study, with the former detected at a concentration of 25.18 mg/g dried weight, approximately ten times higher than the amount reported by Loizzo et al. [20]. Additionally, we quantified flavonols, ellagitannins, and chlorogenic acid in the extract. To assess the antidiabetic potential of *R. ulmifolius* fruit extract, we evaluated its ability to inhibit α-glucosidase and α-amylase, two key enzymes involved in postprandial glucose metabolism. In this case, our results showed a high selectivity for α-glucosidase, with an IC_50_ of 2.8 µg/mL, more than 32 times lower than that of acarbose, a standard glucosidase inhibitor. In this scenario, amylase activity remains unchanged, leading to the breakdown of polysaccharides. On the other hand, inhibiting glucosidase slows down the digestion of oligosaccharides which result from amylase activity. This, in turn, improves postprandial glycemic control. As a result, the absorption of glucose is delayed, thereby reducing glycemic spikes. Moreover, selective inhibition of α-glucosidase over α-amylase is beneficial for minimizing gastrointestinal side effects commonly associated with non-selective inhibitors like acarbose. Acarbose inhibits the activity of both α-glucosidase and α-amylase. Undegraded polysaccharides in the intestine are broken down by bacteria, leading to increased side effects such as intestinal gas production [34].

Our result contrasts with the findings of Loizzo et al. [20], who reported a significantly lower inhibition (IC_50_ of 55.48 µg/mL), which was approximately 1.5 times higher than that of acarbose. Several factors may account for the observed discrepancies in inhibitory activity and phenolic content across studies. First and foremost, variations in plant material origin may play a crucial role in this sense. Many specialized metabolites involved in therapeutic effects are also known to mediate plant responses to environmental stressors [35]. This is particularly true for antioxidant molecules, as the accumulation of ROS can result from a wide range of biotic and abiotic stressors, potentially leading to cell damage and death [36]. Consequently, the environmental conditions of plant collection sites may significantly affect phytochemical profiles and the associated biological activities. This can be particularly relevant when plants are sourced from challenging environments, such as the Mediterranean region. Environmental conditions such as temperature and sunlight exposure have been shown to influence polyphenol content in wild berries [37]. Furthermore, methodological and analytical variables must be considered, as well as differences in the extraction methods (such as solvent choice, extraction duration and use of fresh versus freeze-dried samples). For instance, prolonged thermal extraction may degrade polyphenols, particularly anthocyanins [38], while pH levels have a significant impact on anthocyanin stability, influencing their color and chemical structure [39].

Regarding the activity of cyanidin 3-*O*-glucoside and pelargonidin-3-*O*-glucoside in inhibiting α-glucosidase, this has been previously described in the literature [40,41]. However, both compounds are reported to have a lower inhibition activity than that of acarbose, with IC_50_ values approximately 1.6 times higher. It is important to note that, although these compounds demonstrated an inhibitory effect, their concentration in our extract and their weaker inhibition cannot fully explain the strong α-glucosidase inhibitory activity that we described for the raw fruit extract.

It is plausible that other minor constituents in the extract contributed synergistically to the observed bioactivity. Among the others, ellagitannins have been described for their inhibitory properties towards α-glucosidase [42]. As a result, foods enriched with these compounds have been proposed for use in diabetes management [30,43,44]. Similarly, flavonols were extensively investigated for their inhibition of glucosidase activity contributing to their recognized biological benefits [45].

Therefore, further investigation into the broader chemical composition and the potential synergistic effect within the extract will be crucial to better understanding the mechanisms behind the potent α-glucosidase inhibitory activity emerging from our study.

Interestingly, *R. ulmifolius* fruit extract was able to dose-dependent inhibit the formation of amyloid plaques composed of IAPP, potentially providing a multi-target strategy for T2D treatment. In fact, recent studies have revealed a complex interplay between chronic hyperglycemia, oxidative stress, and misfolding of IAPP, which together contribute to β-cell dysfunction in T2D. Hyperglycemia increases mitochondrial production of ROS and promotes non-enzymatic glycation, leading to advanced glycation end-products. They can activate the receptors for advanced glycation end-products on β-cells, triggering inflammatory cascades and further oxidative stress. This disrupts proteostasis, impairing protein folding and degradation, and it promotes the formation of toxic IAPP oligomers and fibrils, accelerating β-cell stress and apoptosis [46].

Compounds and extracts which reduce glycemic load (e.g., via α-glucosidase inhibition) and block IAPP aggregation may offer synergistic benefits. By lowering postprandial glucose, α-glucosidase inhibitors reduce metabolic stress. Combined with agents that stabilize IAPP or inhibit aggregation, this dual strategy could better preserve β-cell function and slow T2D progression. Considering the composition of the extract, a contribution to the observed inhibitory effect may be attributed to the presence of cyanidin 3-*O*-glucoside, one of the most abundant compounds, which has been reported to increase cell viability and inhibit amyloid formation in human islets exposed to amylin [47]. Among the most studied compounds, epigallocatechin 3-gallate, observed in high amounts in green tea, and curcumin, a polyphenolic natural compound derived from the *Curcuma longa* plant, have also been shown to reduce IAPP aggregation [48]. Currently, there are no clinically approved drugs available to inhibit IAPP aggregation, highlighting the need to identify natural compounds or mixtures with anti-amyloidogenic properties.

In addition, we evaluated the effect of the extract on Caspase-1 activity, an inflammatory protease involved in the maturation and release of pro-inflammatory cytokines such as IL-1β and IL-18. Caspase-1 activation is a central event in inflammasome signaling, and it plays a pivotal role in chronic low-grade inflammation, a well-recognized contributor to the pathogenesis of metabolic diseases such as T2D [4]. Notably, the extract reduced Caspase-1 activity in both intracellular and extracellular compartments, suggesting that *R. ulmifolius* fruits may exert anti-inflammatory effects at the epithelial barrier level. A limitation of the present study is that our cellular model, while useful to explore direct effects on intestinal epithelial cells, does not replicate the complexity of the intestinal microenvironment, including cross-talk with immune cells and the presence of inflammatory mediators. Future studies should therefore extend these observations to more physiologically relevant systems, such as co-cultures or in vivo models, to better capture the integrated anti-inflammatory and immunomodulatory potential of the extract. In parallel, particular attention will be given to evaluating caspase-1 activation in the presence of well-known inflammatory stimuli, such as lipopolysaccharide (LPS) and adenosine triphosphate (ATP). Using these stimuli will allow us to better assess the potential anti-inflammatory effects of the extract under conditions that more closely mimic physiological inflammatory responses.

Overall, these findings highlight the potential of the extract to act through a combination of mechanisms. Indeed, one of the main strengths of this work is the identification of a natural extract capable of targeting multiple pathways involved in diabetes, thus offering a broader therapeutic potential. Traditional therapies often focus on a single aspect of the disease, such as lowering blood glucose levels. However, the multifaceted nature of T2D, encompassing insulin resistance, oxidative stress, and β-cell dysfunction, requires a more comprehensive approach. The *R. ulmifolius* fruit extract appears to be promising in this sense by targeting multiple disease pathways simultaneously: it inhibits carbohydrate-digesting enzymes, prevents amyloid aggregation, reduces oxidative stress, exerts anti-inflammatory effects and is non-toxic to cells, providing compelling evidence of its potential as a functional food for T2D management. The nutritional potential of *R. ulmifolius* fruits has already been described due to their content of bioactive compounds [16]. The findings presented in this study further support and add value to this perspective by providing additional evidence of potential health-promoting properties of *R. ulmifolius*, especially in the prevention of several diseases, such as T2D. *R. ulmifolius* fruit extract could pave the way for new strategies in diabetes care, harnessing the power of nature to address the complex pathophysiology of the disease while promoting overall metabolic health.

## 4. Materials and Methods

### 4.1. Reagents and Standards

All the chemicals used were of analytical grade. Methanol, 85% *w*/*w* phosphoric acid and dimethyl sulfoxide (DMSO) were purchased from Sigma-Aldrich (Steinheim, Germany). Ethanol 96% was purchased from Carlo Erba reagents (Milan, Italy); LC-MS grade acetonitrile, formic acid, and water were purchased from Merck (Darmstadt, Germany). Standards of cyanidin-3-*O*-glucoside, pelargonidin-3-*O*-glucoside, kaempferol-3-*O*-rutinoside, quercetin-3-*O*-glucoside, quercetin-3-*O*-rhamnoside, 5-*O*-caffeoylquinic acid (chlorogenic acid), ellagic acid, and protocatechuic acid were purchased from Extrasynthese (Genay Cedex, France) and TransMIT (Giessen, Germany). Ultrapure water (18 MΩ·cm) was obtained with a Milli-Q Advantage A10 System (Millipore, Milan, Italy).

### 4.2. Plant Material

*R. ulmifolius* fruits were collected in August 2021 from the area of Jerzu (Sardinia, Italy). Botanical identification was carried out by Prof. Cinzia Sanna, and a voucher specimen (CAG 465/b) was deposited in the Herbarium CAG at the University of Cagliari. After collection, fruits were freeze-dried and ground to a fine powder.

### 4.3. Extract Preparation

Fifty grams of fruit powder were extracted three times with 80% ethanol (500 mL per cycle for 24 h each) at room temperature. The combined extracts were subsequently filtered, concentrated under vacuum at 40 °C, and lyophilized to afford 12.7 g of crude extract (yield: 25.4% *w*/*w*).

### 4.4. α-Amylase and α-Glucosidase Inhibition Assay

α-Amylase and α-glucosidase activities were assayed following the methods previously reported [49]. Both activities were determined spectrophotometrically at 405 nm by measuring the amount of 2-chloro-nitrophenol or *p*-nitrophenol released by hydrolysis catalyzed by α-amylase and α-glucosidase, respectively. The inhibitory efficacy was reported as IC_50_ value, indicating the concentration of extract required to suppress 50% of the enzymatic activity. Acarbose, a commercially available antidiabetic agent, served as the standard inhibitor for both enzymes. The type of enzyme inhibition was evaluated using Lineweaver–Burk double reciprocal plots. Kinetic assays were conducted by varying substrate concentrations in the presence and absence of the extract at increasing concentrations. The dissociation constants for the inhibitor binding to the free enzyme (KI) and to the enzyme–substrate complex (KIS) were calculated from the slopes and y-intercepts plotted against extract concentrations, respectively.

### 4.5. ABTS Radical Scavenging Activity

The ABTS (2,2′-azino-bis(3-ethylbenzothiazoline-6-sulfonic acid)) radical scavenging capacity was assessed following the method reported by Delogu et al. [50]. A volume of 10 μL of the extract was mixed with 1 mL of ABTS+ solution previously diluted to reach an absorbance of approximately 0.70 ± 0.05 (mean ± SD). After 1 min of incubation, the absorbance was recorded at 734 nm. Trolox (6-hydroxy-2,5,7,8-tetramethylchroman-2-carboxylic acid) was employed as the reference antioxidant. Results were expressed as the effective concentration required to achieve 50% reduction of the initial absorbance (EC_50_).

### 4.6. Determination of the Total Phenolics and Flavonoids

The total phenolic content of *R. ulmifolius* fruit extract was quantified using the Folin–Ciocalteu method [51]. A 10 µL aliquot of the sample was incubated with 50 µL of Folin–Ciocalteu reagent and 790 µL of distilled water for 1 min, followed by the addition of 150 µL of 20% aqueous sodium carbonate solution. After 45 min of incubation at room temperature in the dark, the absorbance was recorded at 750 nm. The total phenolic content was expressed as milligrams of gallic acid equivalents (GAE) per gram of dried weight extract (dw).

### 4.7. Cell Culture, Cell Viability and Intracellular ROS Levels

Caco-2 cells (American Type Culture Collection ATCC, Manassas, VA, USA) were cultured at 37 °C in a humidified environment with 5% CO_2_, in Dulbecco’s Modified Eagle’s Medium (DMEM, EuroClone, Pero, MI, Italy) supplemented with 10% fetal bovine serum (FBS, Gibco, NY, USA) and 100 Units/mL penicillin/streptomycin.

Cell viability was assessed by the colorimetric 3-(4,5-dimethylthiazol-2-yl)-2,5-diphenyltetrazolium bromide (MTT) assay, as previously described with slight modifications [52]. Metabolically active cells reduce MTT to formazan through dehydrogenase activity, utilizing reducing agents such as NADPH and NADH. The resulting intracellular purple formazan crystals were solubilized and quantified spectrophotometrically.

Cells were seeded into 96-well plates at a density of 10^4^ cells per well. After a 24 h incubation with control or varying concentrations of extracts (0–10 µg/mL), cells were incubated with MTT solution for 3 h at 37 °C. Following the solubilization of formazan in DMSO, absorbance was measured at 590 nm with a reference wavelength of 630 nm using a microplate reader.

Caco-2 cells were first incubated with the extract (0–10 μg/mL) for 24 h, after which they were loaded with10 μM 2′,7′-dichlorofluorescein diacetate (DCFH-DA) for 30 min at 37 °C, and only then briefly exposed to H_2_O_2_ (1 mM) to induce oxidative stress for measuring intracellular ROS production [49]. The fluorescence intensity of DCF was then measured right away using a fluorescent plate reader, which took readings every 5 min for 50 min at an excitation wavelength of 485 nm and an emission wavelength of 530 nm.

### 4.8. Caspase-1 Activity Assay

Caspase-1 enzymatic activity was assessed using the Caspase-Glo^®^ 1 Inflammasome Assay (Promega, Madison, WI, USA) according to the manufacturer’s instructions. In brief, Caco-2 cells were plated in white 96-well plates (Corning, NY, USA) at a density of 2 × 10^4^ cells per well and incubated overnight at 37 °C with 5% CO_2_. After 24 h, cells were exposed to a range of extract concentrations from 0.1 to 100 µg/mL. After 24 h of incubation, two types of measurements were performed.

Cell-associated activity: the assay reagent (containing the Z-WEHD-aminoluciferin substrate and a luminogenic detection buffer) was added directly to the wells containing cells.

Supernatant activity: culture medium was collected, transferred to a clean 96-well plate, and mixed 1:1 with Caspase-Glo^®^ 1 reagent.

Plates were incubated in the dark at room temperature for one hour, after which luminescence (RLU) was recorded using the NIVO™ multimode plate reader (Perkin Elmer, Waltham, MA, USA). All conditions were tested in technical triplicate.

### 4.9. Inhibition of IAPP Aggregate Formation

The primary method to follow the progression of fibril formation in real-time is based on the extrinsic fluorescence of Thioflavin T (ThT), a benzothiazole dye frequently used for the in vitro identification and quantification of amyloid fibrils, including IAPP. The addition of ThT to samples with β-sheet-rich deposits, like the cross-β-sheet quaternary structure of amyloid fibrils, results in a characteristic blue shift and heightened fluorescence, with excitation and emission maxima at about 440 and 490 nm, respectively. The fluorescence of amyloid-ThT complex allows for precise quantification of amyloid fibril production because of the stoichiometric and saturable interaction between ThT and amyloid fibrils. Fluorescence intensity values are plotted over time, and the related spectra are measured. Peptide aggregation without addition of any additive was used as the control sample, and any variation from this sample over the time scale may indicate an acceleration or inhibition of the aggregation processes [53].

In order to create IAPP stock solutions, 250 µL (2 mM) of hexafluorisopropanol (HFIP) were used to dissolve 2 mg of synthetic amylin. The stock solution was kept at −20 °C for storage. Before the measurement, all solutions for these investigations were made by mixing IAPP peptide (in lyophilized dry form) with a PBS buffered (1 mM) ThT solution. The final concentration of the IAPP solution was 40 µg/mL. When the extract of *R. ulmifolius* fruits was present, the IAPP solution-to-extract ratio was at 1:10, 1:5, 1:1, 1:0.1, and 1:0.01 by weight (400 µg/mL, 200 µg/mL, 40 µg/mL, 4 µg/mL, 0.4 µg/mL). ThT fluorescence was observed at 480 nm with 440 nm excitation at 37 °C, on an EnVision multimode plate reader (PerkinElmer). Experiments were conducted in sextuplicate and were repeated three times.

### 4.10. High-Resolution LC-ESI-QToF-MS-MS and HPLC-PDA Analysis

The qualitative characterization of the plant extracts was performed using the method reported by De Luca et al. [54], with some modifications. Briefly, the analytical setup included an advanced ion mobility QToF LC/MS system equipped with a 1290 Infinity II UPLC and a 6560 IM-QToF (Agilent Technologies Inc., Palo Alto, CA, USA) and analyses were performed using an electrospray ionization (ESI) source, operating in negative and positive ion mode. Data were acquired and processed using the Agilent MassHunter Workstation Acquisition software v. B.09.00. (Agilent Technologies). ESI/QToFMS data were then analyzed using the MassHunter Workstation Qualitative Analysis software v. 10.0 (Agilent Technologies) and the MassHunter METLIN metabolite PCDL database v. B.08.00 (Agilent Technologies) and Sirius^®^ software v. 4.7.4 were used for the tentative identification of the metabolites and to predict fragmentation and molecular formulae [22,23], along with comparing experimental MS/MS spectra with fragmentation patterns reported in the literature or with spectra reported in a public repository of mass spectral data. Quantification of targeted phenolic compounds was carried out using an HPLC-PDA method as described by De Luca et al. [55] using an Agilent 1260 Infinity II HPLC system and an Agilent G4212B photodiode array detector (Agilent Technologies). The separation was achieved using a Luna C18 column (150 × 4.60 mm, 2.6 μm, Phenomenex, Castel Maggiore, BO, Italy) with a mobile phase composed of 0.22 M phosphoric acid and acetonitrile properly mixed in gradient elution, at a constant flow rate of 0.8 mL/min. A 10 μL injection volume was used. The chromatograms and spectra were elaborated with an OpenLab V. 2.51 data system (Agilent Technologies), and phenolic compounds were detected and quantified according to the main classes: anthocyanins at 520 nm, flavonols at 360 nm, hydroxycinnamic acids at 313 nm, flavan-3-ols at 210 nm and hydroxybenzoic acids at 280 nm. For the quantitative analysis, plant extracts were initially solubilized with an MeOH:H_2_O 80:20 *v*/*v* mixture (plant—solvent ratio 1:50 *w*/*v*) and diluted 1:1 *v*/*v* with 0.22 M H_3_PO_4_. The solutions were filtered with a 0.22 μm CA syringe filter prior to injection. Phenolic content was expressed as mg/g dw.

### 4.11. Statistical Analysis

Data are expressed as mean ± standard deviation (SD). One-way ANOVA and Tukey’s post hoc test were performed for groups comparison using GraphPad Prism software v. 8 (San Diego, CA, USA). A *p*-value of less than 0.05 was considered statistically significant.

## 5. Conclusions

This study provides evidence for the potential of *R. ulmifolius* fruit extract as a functional food for managing T2D. The extract proved to be a multitarget agent capable of contributing to glycemic control through enzyme inhibition and protecting pancreatic β-cells from amyloid toxicity and oxidative stress. Various bioactive compounds, including anthocyanins, ellagitannins and flavonols, likely contribute to these beneficial effects, aligning with the traditional use of this plant in Mediterranean medicine.

In addition, *R. ulmifolius* fruit extract demonstrated antioxidant activities, reducing ROS production in Caco-2 cells. This suggests that the extract not only assists in controlling glucose levels but also mitigates oxidative stress, a significant contributor to the complications associated with T2D. Moreover, the extract showed the ability to reduce caspase-1 activity in intestinal epithelial cells, indicating a possible role in modulating chronic low-grade inflammation, a known feature of T2D pathophysiology. The absence of cytotoxic effects in cell viability assays supports the potential of *R. ulmifolius* fruits as a functional food. Further investigation into the broader chemical composition and the potential synergistic effect within the extract will be crucial to better understanding the mechanisms behind the potent α-glucosidase inhibitory activity emerging from our study. Moreover, future research will include in vivo studies to test the efficacy of the potential hypoglycemic extract by measuring glycemic levels in control and diabetic rats before and after the administration of a sucrose-loading solution.

## Figures and Tables

**Table 1:** Inhibition of *R. ulmifolius* fruit extract on α-glucosidase and α-amylase activities.

Sample	α-Glucosidase		α-Amylase	
	Inhibition % (100 µg/mL)	IC_50_ (µg/mL)	Inhibition % (100 µg/mL)	IC_50_ (µg/mL)
*R. ulmifolius* fruit extract	99.8	2.80 ± 0.56	8.5	>100
Acarbose		90.0 ± 7.3		8.04 ± 0.65

IC_50_ value represents the mean ± standard deviation for three independent assays.

**Table 2:** Determination of antioxidant activity, total phenolic and flavonoid content of *R. ulmifolius* fruit extract.

Sample	ABTS EC_50_ (µg/mL)	Total Phenolic mg GAE/g dw	Flavonoid mg QE/g dw
*R. ulmifolius* extract	72.42 ± 0.62	28.69 ± 0.92	5.12 ± 0.68
Trolox	3.4 ± 0.3		

Values represent the mean ± standard deviation for three independent assays.

**Table 3:** Concentration of targeted phenolic compounds detected in *R. ulmifolius* fruit extract (mg/g of dried weight (dw), mean ± SD; n = 3).

Compound	No ^§^	*R. ulmifolius* Fruit Extract (mg/g dw)	
		Mean	±SD
**Total Anthocyanins**		29.16	±2.04
cyanidin-3-*O*-glucoside	3	25.18	±1.76
pelargonidin-3-*O*-glucoside	4	2.92	±0.18
cyanidin-3-*O*-xyloside ^a^	5	0.39	±0.04
cyanidin-3-*O*-dioxalyl-glucoside ^a^	6	0.67	±0.03
**Total Flavonols**		0.74	±0.07
quercetin-HMG-glucoside ^b^	10	0.36	±0.04
kaempferol derivative ^c^	11	0.38	±0.02
**Total Ellagitannins**		2.04	±0.16
ellagic acid pentoside ^d^	7	0.69	±0.03
ellagic acid glucuronide ^d^	9	1.35	±0.07
**Total Hydroxycinnamic acid**		0.77	±0.05
chlorogenic acid	2	0.77	±0.05
**Total polyphenols**		32.71	±2.29

^a^ Expressed as cyanidin-3-*O*-glucoside equivalents; ^b^ expressed as quercetin-3-*O*-glucoside equivalents; ^c^ expressed as kaempferol-3-*O*-glucoside equivalents; ^d^ expressed as ellagic acid equivalents; § peak number as reported in Table S1.

## Data Availability

All data are presented in this report.

## Supplementary Materials

The following supporting information can be downloaded at: https://www.mdpi.com/article/10.3390/plants14213247/s1, Figure S1. MTT assay of cells pre-treated with extract and exposed to oxidative stress. Cells were treated with different concentrations of the extract for 24 h, followed by exposure to H_2_O_2_ (2 mM) for 1 h. Cell viability was then determined using the MTT assay. NT: non-treated cells; T: cells treated with H_2_O_2_ only. Data are expressed as a percentage of control. Data represent the mean (±standard deviation) of six independent experiments. All samples are significantly different compared to both NT and T samples (*p* < 0.001). Table S1: Compound identification by (HR) LC-ESI-QTOF MS/MS in *R. ulmifolius* fruit extract.

## Abbreviations

The following abbreviations are used in this manuscript:

T2D	Type 2 diabetes
IAPP	Islet amyloid polypeptide
ThT	Thioflavin T
ROS	Reactive oxygen species
ABTS	2,2′-Azinobis-(3-Ethylbenzothiazoline-6-Sulfonic Acid)
GAE	Gallic Acid Equivalent
QE	Quercetin Equivalent
dw	Dried weight
MTT	3-(4,5-dimethylthiazol-2-yl)-2,5-diphenyltetrazolium bromide assay
DCFHDA	2′,7′-dichlorofluorescein diacetate
HPLC-PDA	High-Performance Liquid Chromatography/Photodiode Array Detector
LC-MS	Liquid Chromatography–Mass Spectrometry
TCC	Total Compound Chromatogram
DMSO	Dimethyl sulfoxide
DMEM	Dulbecco’s Modified Eagle’s Medium
FBS	Fetal Bovine Serum
