# Supplementary material for: Inhibition of α-Glucosidase Activity and Islet Amyloid PolyPeptide Fibril Formation by Rubus ulmifolius Fruit Extract: A Potential Therapeutic Approach for Type 2 Diabetes Mellitus
Source: Plants (Basel). 2025 Oct 23;14(21):3247. doi: 10.3390/plants14213247 (PMC12608329; doi:10.3390/plants14213247)
Supplement: Supplementary file 1 [file plants-14-03247-s001.zip › plants-3880797-supplementary.pdf]

**Figure S1.** MTT assay of cells pre-treated with extract and exposed to oxidative stress. Cells were treated with different concentrations of the extract for 24 h, followed by exposure to $H_2O_2$ (2 mM) for 1 h. Cell viability was then determined using the MTT assay. NT: non-treated cells; T: cells treated with $H_2O_2$ only. Data are expressed as a percentage of control. Data represent the mean (± standard deviation) of six independent experiments. All samples are significantly different compared to both NT and T samples ($p < 0.001$).

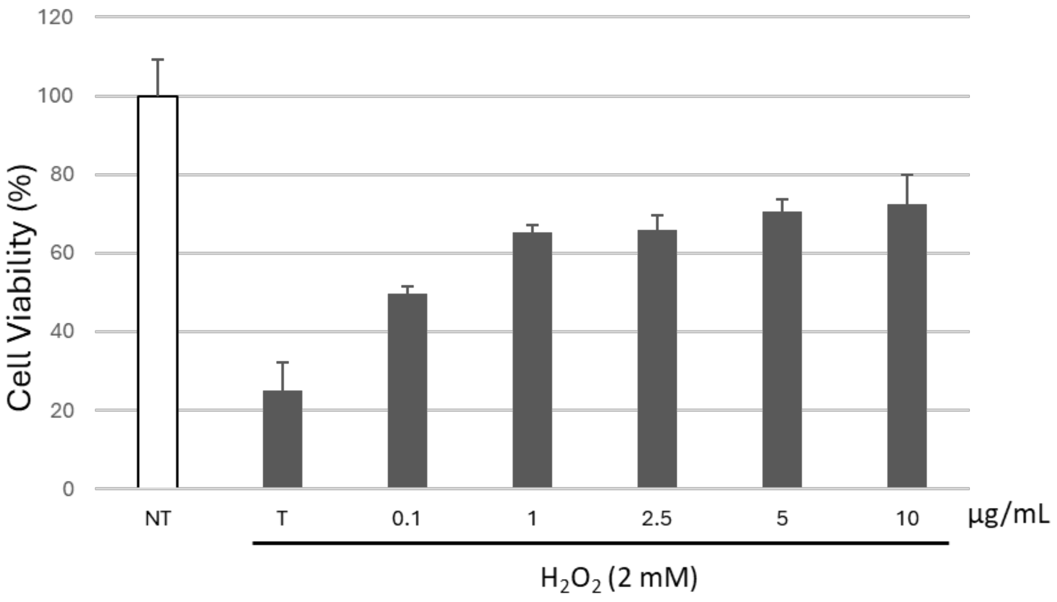


NT = non-treated cells
T = cells treated with $H_2O_2$ only.

**Table S1.** Compounds identification by (HR) LC-ESI-QTOF MS/MS in *R. ulmifolius* fruits extract.

| Compound n° | Rt min | Identity | [M-H]⁻ *m/z* | [M+H]⁺ *m/z* | Molecular formula | Δ ppm | MS/MS* *m/z* | References | Level# |
|---|---|---|---|---|---|---|---|---|---|
| 1 | 4.53 | protocatechuic acid | 153.0443 | | $C_7H_6O_4$ | 6.88 | 153.0443(100) | [1,2] | 1 |
| 2 | 6.44 | chlorogenic acid | 353.110 | | $C_{16}H_{18}O_9$ | -1.11 | 191.0777(100) | [3,4] | 1 |
| 3 | 9.81 | cyanidin-3-*O*-glucoside | | 449.1082 | $C_{21}H_{21}O_{11}^+$ | -0.19 | 287.0548(100) | [3–5] | 1 |
| 4 | 10.9 | pelargonidin-3-*O*-glucoside | | 433.1114 | $C_{21}H_{21}O_{10}^+$ | -0.49 | 271.5544(100) | [3–5] | 2 |
| 5 | 12.07 | cyanidin-3-*O*-xyloside | | 419.0972 | $C_{20}H_{19}O_{10}^+$ | -0.44 | 287.0546(100) | [3–5] | 2 |
| 6 | 12.76 | cyanidin-3-*O*-dioxalyl-glucoside | | 593.2197 | $C_{21}H_{28}O_{15}^+$ | 0.36 | 287.0547(100) | [3–5] | 2 |
| 7 | 16.74 | ellagic acid pentoside | 433.0634 | | $C_{19}H_{14}O_{12}$ | -0.41 | 301.0186(100)/300.0116(30) | [5] | 2 |
| 8 | 17.42 | quercetin-3-*O*-rhamnoside | 447.0774 | | $C_{21}H_{20}O_{11}$ | -1.42 | 300.0128(100)/301.0196(75) | [3,4] | 1 |
| 9 | 18.42 | ellagic acid glucuronide | 477.0849 | | $C_{20}H_{14}O_{14}$ | 0.04 | 301.0560(100) | [5] | 2 |
| 10 | 19.51 | quercetin-HMG-glucoside | 607.1422 | | $C_{27}H_{18}O_{16}$ | -1.91 | 300.0404(100)/301.0549(49) | [3–6] | 2 |
| 11 | 20.24 | kaempferol derivative | 447.1114 | | $C_{21}H_{20}O_{11}$ | -1.65 | 255.0539(100)/284.0536(80)/285.05978(17) | [6–8] | 3 |
| 12 | 28.80 | kaempferol 3-*O*-rutinoside | 593.1423 | | $C_{27}H_{30}O_{15}$ | 1.56 | 284.0541(100)/285.0603(65) | [3,4] | 1 |

* In parenthesis the relative intensity; # according to Blaženović et al. [9]

## References


1. Liu, W.; Nisar, M.F.; Wan, C. Characterization of Phenolic Constituents from *Prunus cerasifera* Ldb Leaves. *J Chem* **2020**, *2020*, 5976090, doi:10.1155/2020/5976090.
2. Truong, X.T.; Park, S.H.; Lee, Y.G.; Jeong, H.Y.; Moon, J.H.; Jeon, T. Il Protocatechuic Acid from Pear Inhibits Melanogenesis in Melanoma Cells. *Int J Mol Sci* **2017**, *18*, doi:10.3390/ijms18081809.
3. Candela, R.G.; Lazzara, G.; Piacente, S.; Bruno, M.; Cavallaro, G.; Badalamenti, N. Conversion of Organic Dyes into Pigments: Extraction of Flavonoids from Blackberries (*Rubus ulmifolius*) and Stabilization. *Molecules* **2021**, *26*, doi:10.3390/molecules26206278.
4. Loizzo, M.R.; Tundis, R.; Leporini, M.; D'urso, G.; Candela, R.G.; Falco, T.; Piacente, S.; Bruno, M.; Sottile, F. Almond (*Prunus dulcis* cv. *casteltermini*) Skin Confectionery By-Products: New Opportunity for the Development of a Functional Blackberry (*Rubus ulmifolius* Schott) Jam. *Antioxidants (Basel)* **2021**, *10*, doi:10.3390/antiox10081218.
5. da Silva, L.P.; Pereira, E.; Pires, T.C.S.P.; Alves, M.J.; Pereira, O.R.; Barros, L.; Ferreira, I.C.F.R. *Rubus ulmifolius* Schott Fruits: A Detailed Study of Its Nutritional, Chemical and Bioactive Properties. *Food Res Int* **2019**, *119*, 34–43, doi:10.1016/J.FOODRES.2019.01.052.
6. Pavlović, A. V.; Papetti, A.; Zagorac, D.Č.D.; Gašić, U.M.; Mišić, D.M.; Tešić, Ž.L.; Natić, M.M. Phenolics Composition of Leaf Extracts of Raspberry and Blackberry Cultivars Grown in Serbia. *Ind Crops Prod* **2016**, *87*, 304–314, doi:10.1016/j.indcrop.2016.04.052.
7. Kashchenko, N.I.; Olennikov, D.N. Phenolome of Asian Agrimony Tea (*Agrimonia asiatica* Juz., Rosaceae): LC-MS Profile, $\alpha$-Glucosidase Inhibitory Potential and Stability. *Foods 2020, Vol. 9, Page 1348* **2020**, *9*, 1348, doi:10.3390/foods9101348.
8. Renai, L.; Scordo, C.V.A.; Chiuminatto, U.; Ulaszewska, M.; Giordani, E.; Petrucci, W.A.; Tozzi, F.; Nin, S.; Bubba, M. Del Liquid Chromatographic Quadrupole Time-of-Flight Mass Spectrometric Untargeted Profiling of (Poly)Phenolic Compounds in *Rubus idaeus* L. and *Rubus occidentalis* L. Fruits and Their Comparative Evaluation. *Antioxidants* **2021**, *10*, 704, doi:10.3390/antiox10050704.
9. Blaženović, I.; Kind, T.; Ji, J.; Fiehn, O. Software Tools and Approaches for Compound Identification of LC-MS/MS Data in Metabolomics. *Metabolites* **2018**, *8*, doi:10.3390/metabo8020031.
